# Acute dysphagia: A rare initial symptom of lateral medullary syndrome: A case report^☆^

**DOI:** 10.1016/j.amsu.2022.104851

**Published:** 2022-11-08

**Authors:** Farhan Ali, Amraha Zubair, Fatima Nazir, Kashif Ali, Sobia Mansoor

**Affiliations:** a Department of Internal Medicine, Chandka Medical College Hospital, Shah Nawaz Bhutto road, Larkana city, Sindh, 77170, Pakistan; b Department of Internal Medicine, Dow Medical College, Dow University of Health Sciences, Mission Rd, Nanak Wara Nanakwara, Karachi City, Sindh, 74200, Pakistan

**Keywords:** Wallenberg syndrome, Posterior inferior cerebellar artery, Stroke, Trauma, Dysphagia, Case report

## Abstract

**Introduction and importance:**

A unique etiology of stroke, lateral medullary syndrome (LMS), is a consequence of posterior inferior cerebellar artery or vertebral artery thromboembolic conditions. LMS patients present particularly with ipsilateral hyperalgesia, ipsilateral ataxia, and Horner's syndrome. Our case signifies that neurogenic origin should always be considered in the absence of local causes of dysphagia. Early diagnosis could prevent LMS complications, including neurological disabilities. A scarcity of research related to dysphagia in LMS, and its outcomes exists. Therefore, the objective is to investigate the clinical course in a patient afflicted with severe dysphagia following a diagnosis of (LMS). This would encourage further research, thus improving management and treatment strategies.

**Case presentation:**

We report a case of a 45-year-old male, a smoker for 20 years, who presented with a single, unique complaint of acute dysphagia for 9 days. According to our knowledge, this is among very few reported cases of LMS with dysphagia being the rare initial complaint. The neurological issues associated with dysphagia gradually improved with the administration of antiplatelet; clopidogrel and lipid-lowering drug; rosuvastatin and the patient was discharged. Atypical presentation in LMS could be supported by the presence of lateral medullary infarct which was confirmed by MRI (Magnetic Resonance Imaging).

**Clinical discussion:**

Dysphagia is a common complaint in multiple gastrointestinal (GI) settings. However, in cases where the GI causes are excluded, as described here, diagnosis of LMS becomes tough.

**Conclusion:**

The diagnosis of LMS was queried owing to the presentation of the single most important common symptom, with no other characteristic manifestations of LMS.

## Highlights

• Lateral Medullary Syndrome (LMS) is caused by thromboembolic occlusive conditions.
• LMS affects the swallowing centers in the dorsolateral medulla, like nucleus ambiguous and reticular formation.
• Dysphagia, the triad of Horner's syndrome, vertigo, and hypoalgesia are among the classical LMS features.
• Single and early presentation of acute dysphagia is a rare presentation of LMS.
• An acute infarct in the left lateral medulla on MRI confirmed the diagnosis of LMS.

## 1 Introduction

In 1808, Gaspard Vieusseux became the first individual to define LMS. Additionally, in 1985 and 1901, through clinical and autopsy findings respectively, Wallenburg further highlighted LMS, thus adding value to the medical literature [1]. Occlusive conditions, including embolism or atherothrombosis, affect posterior inferior cerebellar and vertebral arteries, leading to LMS. Also, rarely Ehler-Danlos Syndrome, fibromuscular dysplasia, and neck manipulation causing dissection of vertebral arteries contribute to the development of LMS [2].

The characteristic manifestations include vertigo, nausea, skew deviation, nystagmus, and ipsipulsion due to vestibular-cerebellar damage [3,4]. Moreover, dysphagia, dysphonia, and dysarthria are caused by nucleus ambiguus pathology [4]. Other detrimental symptoms include ipsilateral facial pain, hypoalgesia, and thermoanesthesia from trigeminal nerve involvement (crossed brainstem syndrome), and hemisensory loss on the contralateral trunk and extremities from spinothalamic tract injury, with hypothalamo-spinal fibers lesions disrupting the sympathetic nervous system giving rise to Horner's syndrome [[4], [5], [6]]. Dysphagia is clinically significant because it is associated with aspiration pneumonia, malnutrition, increased mortality, and long hospital stay [6,7].

The management and treatment strategies implied for dysphagia in LMS incorporate general therapeutic approaches aimed toward dysphagia treatment concerning stroke patients. Also, manifold modern techniques have now been employed globally [7].

Here we will report a case of a 45-year-old male, a smoker for 20 years, having no co-morbid conditions, such as diabetes mellitus or hypertension. He was presented in the outpatient department with an unusual complaint of sudden acute dysphagia for nine days. Possible causes of dysphagia were ruled out, except for the neurogenic cause which was confirmed by MRI brain. The early presentation of dysphagia in the disease course, with no other associated symptoms, is an atypical feature in our case. Thus, questioned the diagnosis of a simple LMS.

The case report has been reported in line with the SCARE criteria [8].

## 2 Case presentation

A 45-year-old male, a known smoker for 20 years, self-presented in Chandka Medical College teaching hospital's OPD with the chief complaint of sudden onset acute dysphagia for 9 days, both for solids and liquids. According to the patient's attendant, he would regurgitate anything he eats. It was not associated with hiccups, hoarseness of voice, weight loss, chest pain, halitosis, and dyspnea. He had no past surgical history, while the past medical history revealed that he was admitted to the psychiatry ward due to an unknown psychiatric illness 20 years back. Furthermore, his family history revealed no significant information. Additionally, he was not taking any sort of medication and no proven allergies were reported.

On general physical examination, he seemed healthy, and Glasgow Coma Scale **(**GCS) was 15/15. The vital signs examination revealed a regular heartbeat of 88 beats per minute, a non-invasive blood pressure of 180/110 mmHg, a temperature of 98.6F, and a respiratory rate of 16 breaths per minute. While pulse oximeter showed 99% saturation on room air, and random blood sugar was 114mg/dl. Moreover, a detailed neurological examination revealed that there was an absent gag reflex and deviation of the uvula towards the right side, whereas all other cranial nerves were intact. The symptoms of vertigo, gait disturbance, ipsilateral Horner syndrome, ipsilateral dysmetria, dysrhythmia, spontaneous nystagmus, ipsilateral facial pain, and contralateral loss of pain and temperature sensation were not reported.

Initial lab investigations showed an elevated white blood cell (WBC) count of 12.6 × 10^9^/L, elevated neutrophil percentage of 80%, increased erythrocyte sedimentation rate (ESR) of 28mm/hr, decreased lymphocyte percentage of 16%, and an increased platelet count of 510 × 10^9^/L. [Table 1]

**Table 1:** Shows the lab values.

TEST	RESULT	REFERENCE RANGE
Total leukocyte count ( × 10^9^/L)	12,600	4–11
Neutrophil percentage	80	50–75
Lymphocyte percentage	16	20–50
Platelet count ( × 10^9^/L)	510,000	150–400
Hemoglobin (g/dL)	14.8	13–18
Monocyte percentage	03	2–8
Eosinophil percentage	01	1–4
Premature cells percentage	No immature cells seen	0–1
Erythrocyte Sedimentation Rate	28	3–15

**Fig. 1:**
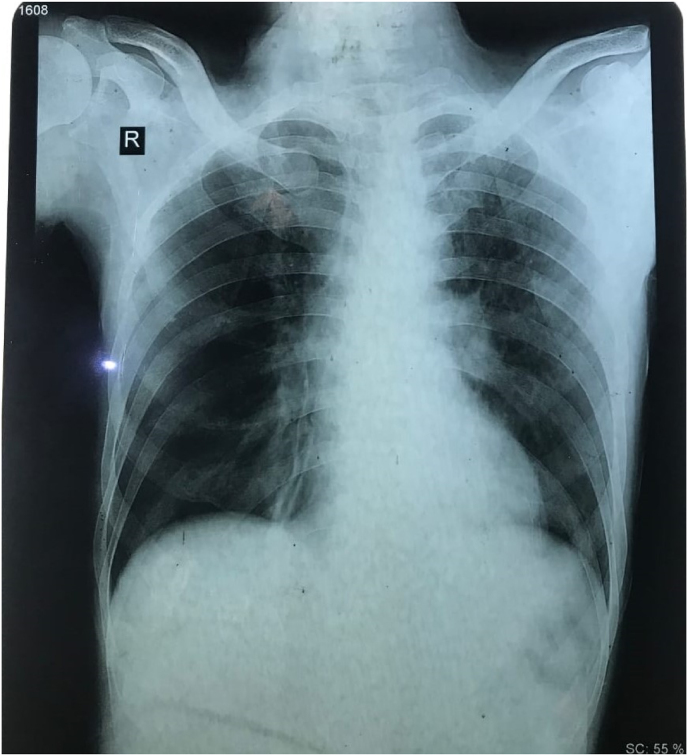
Chest x-ray revealing no significant findings; normal.

**Fig. 2:**
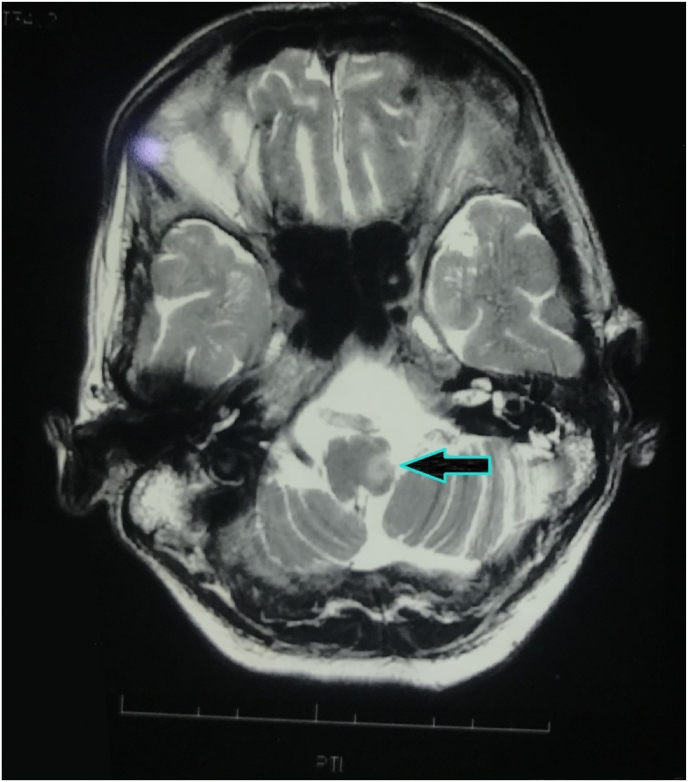
MRI Brain showing infarct in left lateral medulla hypodense area (pointing arrow) (without contrast).

Initially, we suspected the local causes of dysphagia. These included not only the structural benign lesions, such as esophageal strictures, Schatzki ring, and esophageal web, but also malignant conditions, like esophageal adenocarcinoma, squamous cell carcinoma, and extrinsic compression. Furthermore, motility disorders, including diffuse esophageal spasm, and achalasia were also presumed. Therefore, an upper gastrointestinal endoscopy was performed on the third day of hospital admission, which showed right vocal cord paralysis with no masses. Thereby, excluding the local causes. Also, the Chest X-ray revealed no significant findings [Fig. 1]. This led us to investigate the neurogenic cause. Therefore, an MRI brain was done which confirmed the presence of an acute infarct in the left lateral medulla, and the lateral medullary syndrome diagnosis was established [Fig. 2]. Electrocardiography and echocardiography findings were within normal limits.

The management and treatment strategies during his hospital stay comprise nasogastric feeding tube, antibiotic ceftriaxone twice a day, injection (proton pump inhibitor) 40 mg intravenously once a day, injection dextrose 10% intravenously once a day, injection ringer lactate intravenously once a day (fluid resuscitation), injection normal saline 0.9% intravenously once a day. Additionally, antiplatelet therapy (clopidogrel) and lipid-lowering drug (rosuvastatin) were administered to mitigate the risk of another stroke. He recovered gradually over the next week; improvement for solids was significant. Therefore, he was discharged, but had not shown up for a follow-up.

## 3 Discussion

Dysphagia is noteworthy among the classical manifestations of vertebral artery or posterior inferior cerebellar artery obstruction [7]. It is widespread, because of mechanical obstruction, dysmotility or neurologic disease especially brainstem infarction of the swallowing centers in the rostral dorsolateral medulla which occurs in lateral medullary infarction (LMI) [9]. In a series of 123 consecutive patients with LMS documented by angiography, vertebral artery disease was present in 67% whereas posterior inferior cerebellar artery disease in 10% population [10]. In India, a study revealed that 22.3% of posterior circulation stroke patients experience dysphagia [11]. Dysphagia in LMS includes impaired coordination during pharyngeal and esophageal phases of swallowing due to the involvement of swallowing centers in the dorsolateral medulla oblongata that are nucleus ambiguous, nucleus tractus solitarius, and the reticular formation [9]. Wallenberg's syndrome is characteristically presented with vertigo, dysphagia, ipsilateral ataxia, decreased facial sensation, Horner's syndrome, and decreased sensation on the contralateral body [5]. However, the pattern of signs and symptoms fluctuates according to the site of the lesion. Severe dysphagia can complicate the clinical picture in 40% of patients with WS [9]. Atypical single feature in our case, which questioned the diagnosis of a simple LMS, was acute dysphagia, with the absence of other classical symptoms of LMS. Dysphagia is not the key symptom at beginning of Wallenberg's syndrome, so this case relates to an unusual presentation of this disease [9]. Dysphagia is extra prominent and lasts longer in WS patients than in hemispheric stroke patients [9]. LMS remains an unidentified reason for dysphagia in the clinical practice of the gastroenterologist. In the majority of WS patients, dysphagia is initially severe enough to require non-oral feeding but often spontaneously recovers within 1–2 months following stroke [9]. In our case, we set up an NG (nasogastric) feeding at the beginning, and then a progressive rehabilitation of food intake was established until recovered. The unavailability of Fiberoptic Endoscopic Evaluation of Swallowing (FEES) and VitalStim therapy for stroke has been the limitation of this study. Therefore, we urge that recent diagnostic and treatment techniques should be employed. Accordingly, repetitive transcranial magnetic stimulation; a non-invasive procedure, and invasive interventions, like balloon catheter dilatation, myotomy for relaxation of the cricopharyngeal muscle, and botulinum toxin injection are among the broad treatment options [7].

## 4 Conclusions

We present an unusual case of LMS, in which dysphagia was the main symptom at the onset. The neurogenic origin of acute oropharyngeal dysphagia should be presumed in the practice of gastroenterology especially when gastrointestinal endoscopy is normal.

## Ethical approval

Not applicable.

## Sources of funding

None.

## Author contribution

Farhan Ali provided the unique case and all the associated information and figures. Amraha Zubair, Fatima Nazir, Kashif Ali, and Sobia Mansoor wrote, formatted, designed, edited, and proofread the content of the manuscript.

## Conflict of interest

There are no conflicts of interest.

## Research registration

It is a case report, so this registration was not required.

## Guarantor

Farhan Ali, Amraha Zubair, Fatima Nazir, Kashif Ali, Sobia Mansoor.

## Provenance and peer review

Not commissioned, externally peer reviewed.

## Consent of patient

Written informed consent was obtained from the patient for publication of this case report and accompanying images. A copy of the written consent is available for review by the Editor-in-Chief of this journal on request.

## Financial support and sponsorship

None.

## Disclosure

The authors hereby certify that the work shown here is genuine, original, and not submitted anywhere, either in part or full. All the necessary permissions from the patient, hospital and institution have been taken for submitting patient's information.

## References

[1] Renjen P., Krishnan R., Di Chaudhari, Ahmad K. (2021). An atypical presentation of left lateral medullary syndrome - a case report. Neurol. India.

[2] Shetty S.R., Anusha R.L., Thomas P.S., Babu G.S. (2012). Wallenberg's syndrome. J. Neurosci. Rural Pract..

[3] Paliwal V.K., Kumar S., Gupta D.K., Neyaz Z. (2015). Ipsipulsion: a forgotten sign of lateral medullary syndrome. Ann. Indian Acad. Neurol..

[4] Gasca-González O.O., Pérez-Cruz J.C., Baldoncini M., Macías-Duvignau M.A., Delgado-Reyes L. (2020). Neuroanatomical basis of Wallenberg syndrome. Cir. Cir..

[5] von Heinemann P., Grauer O., Schuierer G., Ritzka M., Bogdahn U., Kaiser B. (2009). Recurrent cardiac arrest caused by lateral medulla oblongata infarction. BMJ Case Rep..

[6] Battel I., Koch I., Biddau F., Carollo C., Piccione F., Meneghello F. (2017). Efficacy of botulinum toxin type-A and swallowing treatment for oropharyngeal dysphagia recovery in a patient with lateral medullary syndrome. Eur. J. Phys. Rehabil. Med..

[7] Jang S.H., Kim M.S. (2021). Dysphagia in lateral medullary syndrome: a narrative review. Dysphagia.

[8] Agha R.A., Franchi T., Sohrabi C., Mathew G., Kerwan A., Thoma A. (2020). The SCARE 2020 guideline: updating consensus surgical CAse REport (SCARE) guidelines. Int. J. Surg..

[9] el Mekkaoui A., Irhoudane H., Ibrahimi A., Yousfi M el (2012). Dysphagia caused by a lateral medullary infarction syndrome (Wallenberg's syndrome. Pan Afr. Med. J..

[10] Loaeza-del Castillo A., Barahona-Garrido J., Criales S., Chang-Menéndez S., Torre A. (2007). Wallenberg's syndrome: an unusual case of dysphagia. Case Rep. Gastroenterol..

[11] Gupta H., Banerjee A. (2014). Recovery of Dysphagia in lateral medullary stroke. Case Rep. Neurol. Med..

